# Clinical Approach to Nosocomial Bacterial Sepsis

**DOI:** 10.7759/cureus.28601

**Published:** 2022-08-30

**Authors:** Pramod Reddy

**Affiliations:** 1 Internal Medicine, University of Florida College of Medicine, Jacksonville, USA

**Keywords:** culture negative sepsis, extended spectrum beta lactamases, beta-lactamase enzymes, septic shock, sepsis

## Abstract

Bacterial sepsis and septic shock are associated with a high mortality, and when clinically suspected, clinicians must initiate broad-spectrum antimicrobials within the first hour of diagnosis. Thorough review of prior cultures involving multidrug-resistant (MDR) pathogens along with other likely pathogens should be performed to provide an appropriate broad-spectrum empiric antibiotic coverage. The appropriate antibiotic loading dose followed by individualized modification of maintenance dose should be implemented based on the presence of hepatic or renal dysfunction. Use of procalcitonin is no longer recommended to determine need for initial antibacterial therapy and for de-escalation. Daily reevaluation of appropriateness of treatment is necessary based on the culture results and clinical response. All positive cultures should be carefully screened for possible contamination or colonization, which may not represent the true organism causing the sepsis. Culture negative sepsis accounts for one-half of all cases, and de-escalation of initial antibiotic regimen should be done gradually in these patients with close monitoring.

## Introduction and background

Sepsis is defined as life-threatening organ dysfunction caused by a dysregulated host response to infection. Systemic inflammatory response syndrome (SIRS) is no longer included in the definition since it is not always caused by an infection. Sepsis occurs due to the release of proinflammatory mediators in response to an infectious disease (bacteria, viruses, fungi, or protozoa). The resulting endothelial injury results in hypotension (shock) due to a combination of increased vascular permeability and release of vasodilatory substances (prostacyclin, nitric oxide). Sepsis is also associated with a decrease in the number of functional capillaries (impaired oxygen extraction) and can affect any organ system in the body. Organ-specific manifestations of sepsis (irrespective of the source of infection) typically involve lungs (tachypnea, acute respiratory distress syndrome), gastrointestinal tract (ileus, translocation of bacteria and endotoxins), liver (transaminitis, hyperbilirubinemia), kidney (acute tubular necrosis) and nervous system (altered mentation, encephalopathy, delayed peripheral neuropathy). Due to unclear reasons, patient mortality is increased when sepsis is associated with acute renal failure.

## Review

The clinical presentation of sepsis ranges from early sepsis (infection and bacteremia) to sepsis (organ dysfunction) and septic shock (persistent hypotension and lactic acidosis despite adequate fluid resuscitation). In most hospitalized patients, sepsis is of bacterial origin (around 70%), and gram-negative bacilli are frequently implicated in patients with severe disease (e.g., septic shock). In approximately one-half of cases of sepsis, an organism is not identified, which is termed “culture negative sepsis” [1]. Sepsis results in mortality rates of around 10% in patients with sepsis (SOFA score >2) and more than 40% in patients with septic shock [2]. Older patients ≥ 65 years of age account for the majority (60 to 85%) of all episodes of sepsis. Other patient characteristics that make them prone to sepsis include diabetes, obesity, renal failure, liver failure, malignancy, HIV, and immunosuppressant medications. Previous hospitalizations are associated with a three-fold increased risk of developing sepsis in the subsequent 90 days [3]. The incidence of sepsis is highest in the winter months and varies among different racial and ethnic groups, with the highest incidence in African-American males [4]. The most common bacterial infections encountered in hospitalized patients occur in the lungs (pneumonia, empyema), abdomen (colitis, cholangitis), skin (cellulitis, fasciitis), central nervous system (meningitis), kidneys, and urinary bladder (urinary tract infections, UTI).

The initial workup of septic patients

For patients with sepsis and septic shock, therapeutic priorities include establishing vascular access (two IV lines) for early administration of fluids and antibiotics. Along with routine laboratory studies (complete blood count with differential, complete metabolic panel, C-reactive protein, serum lactate), it is important to obtain two sets of blood cultures (aerobic and anaerobic) from two distinct venipuncture sites and all indwelling vascular access devices. Infusion of intravenous crystalloids (30mL/kg) should be started along with empiric broad-spectrum antibiotic therapy within the first 45 minutes. Efforts should be made to obtain cultures from easily accessible sites (e.g., blood, urine, cerebrospinal fluid, sputum), and imaging of suspected sources (e.g., chest, abdomen) should be ordered [5]. Microbiologic evaluation in patients with suspected pneumonia may also include serologies for influenza, parainfluenza, respiratory syncytial, adenoviruses, and urine antigens for *S. pneumoniae* and *L. pneumophila*.

Use of Biomarkers in Sepsis

Several nonspecific laboratory abnormalities observed in septic patients include leukocytosis, leukopenia, normal WBC count with greater than 10% immature/Band forms, hyperlactatemia, elevated erythrocyte sedimentation rate, C-reactive protein and procalcitonin level. While elevated serum procalcitonin levels are typically associated with bacterial infections, due to poor sensitivity and specificity, 2019 IDSA guidelines no longer recommend the use of procalcitonin in sepsis workup [6].

True Infection Versus Colonization

The human body is host to a vast number of microorganisms (*S. epidermidis*, *S. aureus*, *Enterobacteriaceae*, C*andida Albicans,* and anaerobes), and these organisms may be cultured from many sites in healthy patients without active infection. The patient-specific factors that increase the risk of colonization and nosocomial infections include malnutrition, immunosuppression, skin breakdown, diabetes, and granulocytopenia. Colonization with pathogenic organisms can occur rapidly after hospitalization in the skin, respiratory tract, urinary tract, and gastrointestinal tract. Colonized organism growth can also exceed the 100,000 thresholds (10^5^ CFU/ml) in chronic Foley and tracheal secretions. Many times, distinguishing the true infection from mere colonization is difficult and often results in the overtreatment of patients. In general, colonized patients with MDR pathogens (e.g., Methicillin-resistant Staph Aureus (MRSA), Vancomycin-resistant enterococcus (VRE), and extended-spectrum beta-lactamases (ESBL)) in cultures from the urine and respiratory secretions are clinically asymptomatic and require changing the indwelling device (e.g., Foley catheter). Immunocompromised patients with prior colonization with multidrug-resistant gram-negative organisms are prone to develop subsequent bacteremia [7].

True Bacteremia Versus Contamination

Properly collected blood cultures (at least two sets) are crucial for the diagnosis of bloodstream infections, but their diagnostic value is affected when a microorganism of questionable evidence is isolated [8]. Collecting a single blood culture should always be avoided due to a lack of sensitivity and the ability to distinguish true bacteremia from contaminants. Organisms like coagulase-negative staphylococci, corynebacterium species (diphtheroids), cutibacterium acnes, bacillus, and micrococcus species are usually found to be blood culture contaminants. On the other hand, the presence of organisms like *enterococci*, *staphylococcus lugdunensis*, and *streptococcus viridans* in blood cultures may be either clinically significant in some patients (immunocompromised or IV drug users) or may reflect contamination, and clinical correlation is required. Even when a single blood culture is positive with organisms like *S. aureus*, *streptococcus pneumoniae*, group A *streptococcus*, *Enterobacteriaceae*, *haemophilus influenzae*, *pseudomonas aeruginosa*, *bacteroides,* and *candida species*, it should be considered clinically significant.

Choosing an empiric antibiotic regimen

The choice of antimicrobials should be tailored to each patient based on the history (e.g., use of IV drugs, previous hospitalizations, and organisms), comorbidities (e.g., diabetes, CKD [chronic kidney disease], liver failure), immune defects (e.g., HIV, chemotherapy, neutropenia), suspected site of infection, presence of invasive devices (e.g., central line, hemodialysis catheter) and local resistance patterns. The possibility of multidrug-resistant (MDR) pathogens should be considered in all septic patients with recurrent admissions, and a thorough review of prior culture results along with drug sensitivities should be performed. Initial coverage should be directed against both gram-positive and gram-negative bacteria that commonly cause sepsis, which include *staphylococcus aureus*, *streptococcus pneumoniae,* and *Enterobacteriaceae* (*escherichia coli*, *klebsiella*, *proteus*, *enterobacter*, *serratia*, and *citrobacter*). The administration of intravenous broad-spectrum antibiotics should be initiated within an hour of sepsis recognition to prevent mortality [9]. Appropriate collection of cultures from the blood and the site of infection (e.g., CSF) is essential before starting antimicrobial therapy but should not delay the administration timeline. In septic patients with difficult IV access, administration of IM cefepime should be considered to avoid a treatment delay.

Commonly Used Empiric Antibiotic Regimens in Sepsis

Since MRSA is a potential cause of sepsis in both community-acquired and nosocomial infections, intravenous vancomycin should be an integral part of any broad-spectrum empiric antibiotic therapy. Vancomycin loading dose should be used with a goal trough level of at least 15 mg/L, which is known to result in higher success rates in septic patients [10]. Antipseudomonal cephalosporins (e.g., cefepime 2 grams every eight hours) or B-lactam/β-lactamase inhibitors (e.g., piperacillin-tazobactam 4.5 grams every eight hours) should be used as part of empiric broad-spectrum antibiotic therapy.

Carbapenems (e.g., meropenem) should be chosen over either cefepime or piperacillin-tazobactam in patients suspected to have infections with extended-spectrum β-lactamases (ESBLs), which are released by gram-negative organisms like *klebsiella* and *Escherichia coli*. Risk factors for ESBL infection include prior administration of antibiotics, presence of urinary or vascular catheters, and prolonged hospital or intensive care unit (ICU) stays. The hospital physicians need to appreciate the differences in the antimicrobial spectrum between various commonly used gram-negative antibiotics (Table 1) to choose the appropriate agent for the suspected organism.

Cefepime Versus Piperacillin-Tazobactam

Cefepime has less anaerobic coverage (only covers *Peptostreptococcus*) and lacks *Enterococcus* coverage when compared to piperacillin-tazobactam (Table 1). Nephrotoxicity (incidence 5%-43%) occurs more commonly with the combination of vancomycin and piperacillin-tazobactam compared to the combination of vancomycin and cefepime [11]. It is important to note that the use of piperacillin-tazobactam alone (without vancomycin) only rarely causes nephrotoxicity (<1%). Since each dose of piperacillin-tazobactam contains 250mg of sodium, cefepime is preferred in patients with decompensated heart failure. If simultaneous anaerobic coverage is necessary for these patients, cefepime should be combined with oral metronidazole since intravenous metronidazole contains 600mg sodium per dose.

**Table 1:** Differences in coverage between commonly used gram-negative antibiotics UTI: Urinary tract infection, ESBL: Extended spectrum beta-lactamase

Spectrum	Ceftriaxone	Cefepime	Piperacillin/ Tazobactam	Ampicillin/Sulbactam	Meropenem
Pseudomonas	None	Excellent	Excellent	None	Excellent
*E. Coli*, *Klebsiella*, *Proteus*	Excellent	Excellent	Excellent	Very poor	Excellent
*Enterobacter*, *Citrobacter*, *Serratia*	Avoid use	Excellent	Moderate	Very poor	Excellent
Acinetobacter	Poor	Moderate to poor	Moderate to poor	Excellent	Moderate to poor
ESBL	None	None	Very good for UTI coverage.	None	Excellent
Anaerobes	None	Poor	Excellent	Excellent	Excellent
*Enterococcus faecium* & *faecalis*	None	None	Excellent for E. faecium & faecalis	Excellent for E. faecium & faecalis	Covers only E. faecalis
Listeria	None	None	Excellent	Excellent	Excellent
CSF penetration	Excellent	Excellent	Poor	Excellent	Excellent

Choice of Empiric Antibiotic Agents in Patients With Documented Penicillin Allergy

Most patients with penicillin allergy manifesting either as simple rash or anaphylaxis (e.g., angioedema, intubation) can safely tolerate other beta-lactam antibiotics like cephalosporins, carbapenems, and aztreonam. While cephalosporins can have a unique allergy, carbapenem allergy is extremely rare. In hospitalized patients with sepsis, physicians are encouraged to use either cefepime or meropenem as agents of choice over aztreonam, which has a widespread bacterial resistance [12]. The mortality benefit of providing the proper antimicrobial coverage will outweigh the small risk of an allergic reaction, which can be easily managed in the inpatient setting.

Empiric Coverage for Other Organisms

Anaerobic infections are suspected when there is foul-smelling discharge or when the site of active infection is normally colonized by anaerobes (e.g., colitis). Metronidazole is the drug of choice for the management of bacteroides infections (intra-abdominal abscesses, bacteremia) and brain abscesses. Clindamycin, ampicillin/sulbactam, and amoxicillin/clavulanic acid are ideal agents for treating bite wounds, dental infections, Ludwig’s angina, Lemierre syndrome, aspiration pneumonia, empyema, and lung abscess [13]. Piperacillin/tazobactam or meropenem can be used to treat mixed nosocomial gram-negative and anaerobic infections (e.g., intraabdominal abscess, peritonitis, and Fournier gangrene). The addition of coverage for atypical organisms (e.g., mycoplasma, chlamydia, and legionella) with a course of macrolide/quinolone should be considered in septic patients with community-acquired pneumonia.

Some of the commonly available broad-spectrum gram-negative agents will be discussed below, followed by gram-positive antibiotics.

Broad-spectrum gram-negative antibiotics

Beta-lactam antibiotics (penicillins, cephalosporins, carbapenems, aztreonam) are among the most commonly prescribed drugs in hospitalized patients with sepsis. Beta-lactam antibiotics are bactericidal and work by inactivating the enzymes located in the bacterial cell membrane, known as penicillin-binding proteins (PBPs), which are involved in cell wall synthesis. The major mechanism of resistance to the beta-lactam antibiotics is the production of either chromosomal or plasmid-mediated enzymes (β-lactamases) that cleave penicillins (penicillinases), cephalosporins (cephalosporinases) and carbapenems (carbapenemases). Decreased penetration to the plasma membrane target site and alterations in the Penicillin-binding proteins (PBPs) are other mechanisms of resistance.

β-lactamase Production

Most gram-negative and some anaerobic organisms (bacteroides) produce β-lactamases, which can induce resistance to commonly used β-lactam antibiotics. AmpC β-lactamases are released by many *Enterobacteriaceae* (e.g., *enterobacter*, *serratia*, and *citrobacter*) and confer resistance to third-generation cephalosporins (cefotaxime, ceftazidime, and ceftriaxone), where an isolate initially susceptible to these agents may become resistant upon therapy. Certain broad-spectrum β-lactam antibiotics (e.g., cefepime and carbapenems) are intrinsically resistant to destruction by β-lactamases and others are combined with β-lactamase inhibitors (piperacillin/tazobactam). It is important to note that clinical efficacy is not guaranteed by adding a β-lactamase inhibitor to a particular class of penicillin. For example, adding clavulanic acid to amoxicillin minimally changes the poor efficacy against most gram-negative organisms.

In most hospitalized patients, the β-lactamase production spectrum varies from inducing minimal resistance (e.g., easy to treat with lower generation cephalosporins) to moderate resistance (e.g., requiring cefepime or piperacillin/tazobactam). However, in some cases, klebsiella and *E. coli* can produce extended-spectrum β-lactamases (ESBLs) that typically require therapy with carbapenems. In rare cases, these organisms may also produce carbapenemases (e.g., klebsiella pneumoniae carbapenemase (KPC) or New Delhi metallo β-lactamase (NDM), which are resistant to carbapenem therapy [14]. Carbapenemase-producing strains require treatment with cefiderocol or combination β-lactamase inhibitors like meropenem/vaborbactam (Table 2). The currently available combination agents are not effective against metallo β- lactamases (e.g., NDM), and newer β- lactamase inhibitors (zidebactam, nacubactam) against NDM strain are under investigation in clinical trials.

**Table 2:** Commonly available combination β-lactamase inhibitors ESBL: Extended spectrum beta lactamase; KPC: Klebsiella pneumoniae carbapenemase

Inhibitor	Clavulanic acid	Sulbactam	Tazobactam		Avibactam	Vaborbactam	Relebactam
Β-lactam	Amoxicillin	Ampicillin	Piperacillin	Ceftolazane	Ceftazidime	Meropenem	Imipenem- Cilastatin
US Brand name	Augmentin	Unasyn	Zosyn	Zerbaxa	Avycaz	Vabomere	Recarbrio
Best uses	Otitis media, sinusitis, respiratory tract infections, bite wounds, anaerobes	Same spectrum as augmentin plus enhanced activity against acinetobacter.	Expands the spectrum of piperacillin to include β-lactamase producing S*. aureus*, *haemophilus*, *neisseria*, *Enterobacteriaceae* and *anaerobes* (*bacteroides*).	Broad spectrum activity against pseudomonas and most ESBL producing organisms.	Broad spectrum activity against pseudomonas and most ESBL producing organisms and KPC.	The main use of these agents is for the treatment of KPC-producing Enterobacteriaceae. The addition of vaborbactam or relebactam does not enhance the clinical activity of carbapenems against carbapenem-resistant P. aeruginosa or acinetobacter species.	
Notes	Poor gram-negative coverage due to widespread resistance.	Poor gram-negative coverage with the exception of Acinetobacter.	Combination is not effective for piperacillin-resistant strains of pseudomonas.	Poor gram-positive coverage.	No coverage for acinetobacter species.	Not active against metallo β-lactamases (e.g., New Delhi strain)	

Cephalosporins

Cephalosporins are grouped into five "generations" based on their spectrum of activity, and the newer siderophore cephalosporin (cefiderocol) is being classified as “other cephalosporins” (Table 3). First-generation cephalosporins are commonly used to treat cellulitis, UTI, and MSSA bacteremia. Second-generation cephalosporins (cefoxitin) have anaerobic coverage and can be used to treat pelvic inflammatory disease.

**Table 3:** Differences in coverage between various classes of cephalosporins MRSA: Methicillin resistant *S. Aureus*; ESBL: Extended spectrum beta lactamase; NDM: New Delhi metallo-carbapenemase

Classes	1	2	3	4	5	Other
Examples	Cefazolin Cephalexin	Cefoxitin Cefaclor	Ceftriaxone Cefotaxime Ceftazidime Ceftazidime/Avibactam	Cefepime	Ceftaroline Ceftolozane/ Tazobactam	Cefiderocol
Best Uses	Cellulitis, Urinary tract infections. IV cefazolin for MSSA bacteremia.	The only class with some anaerobic coverage. Pelvic inflammatory diseases (PID)	Community-acquired pneumonia, urinary infections, spontaneous bacterial peritonitis. Ceftazidime-Avibactam covers ESBL.	Excellent pseudomonas and other AMP-C producing organism coverage (except acinetobacter)	Ceftaroline covers MRSA but NOT pseudomonas. Ceftolozane/ Tazobactam covers ESBL, pseudomonas but NOT MRSA. Ceftobiprole covers BOTH MRSA & pseudomonas.	ESBL, *Carbapenemase*, producing organisms, multidrug-resistant *Pseudomonas*, *Acinetobacter,* and *Stenotrophomonas*.
Notes	Avoid use in cerebral infections due to poor CSF penetration		Avoid routine use of ceftazidime due to β- lactamase induction. Ceftriaxone can cause biliary sludge after prolonged administration.	The preferred agent for empiric use.	Ceftaroline can be used synergistically with daptomycin.	Not active against NDM *Carbapenemase*. Poor gram-positive coverage

Third-generation cephalosporins are less active against *staphylococcus* than the first-generation cephalosporins, but highly active against pneumococcal infections (pneumonia, meningitis). Treatment of AmpC-producing Enterobacteriaceae (e.g., *enterobacter*, *citrobacter,* and *serratia*) with third-generation cephalosporins should be avoided since resistance can rapidly emerge during therapy [15]. Ceftriaxone can cause biliary sludge after prolonged administration since 40% of the drug is secreted into the bile and can precipitate with calcium. Patients receiving TPN and recovering from major surgery are more prone to this effect, and the sludge is reversible in most cases after discontinuation of the drug. Around 14% of the sludge-positive patients may develop "ceftriaxone gall stones", which can cause obstructive jaundice and acute cholecystitis [16-17].

Only certain third-generation agents (e.g., ceftazidime, cefoperazone) possess pseudomonas coverage, but they are rarely used empirically due to the fear of inducing β-lactamase production by bacteria. The addition of β-lactamase inhibitor to anti-pseudomonal third-generation cephalosporins (e.g., avibactam to ceftazidime) extends the spectrum of activity to include most *Enterobacteriaceae* (including those that produce AmpC β-lactamase, ESBL, and KPC).

Fourth-generation cephalosporins (cefepime) have greater activity against the AmpC β-lactamase producing gram-negative organisms. Cefepime-induced neurotoxicity (CIN) occurs primarily in patients with renal dysfunction as the antibiotic is primarily renally excreted. The symptoms include altered mental status (decreased consciousness, confusion, encephalopathy, aphasia, myoclonus), nonconvulsive status epilepticus, and tonic-clonic seizures. Cefepime neurotoxicity has a variable incidence (1%-15%) and is preventable in most cases by properly adjusting the dose in patients with renal failure [18]. The currently available fifth-generation cephalosporins have a variable antimicrobial spectrum between ceftaroline (has activity against MRSA but not pseudomonas) and ceftolozane-tazobactam (active against pseudomonas but not MRSA). Ceftobiprole (currently not available in the U.S.) is effective against both pseudomonas and MRSA.

Cefiderocol is a siderophore cephalosporin with activity against most gram-negative bacteria, including ESBL and carbapenemase-producing organisms) and multidrug-resistant *pseudomonas*, *acinetobacter*, *stenotrophomonas* and *burkholderia* [19].

Carbapenems

Carbapenems (imipenem/cilastatin, meropenem, doripenem, ertapenem) are resistant to cleavage by most plasmid and chromosomal β-lactamases with a broad spectrum of activity against β- lactamase producing strains of gram-negative organisms (including ESBL) and anaerobes. Carbapenem resistance among Enterobacteriaceae has been increasing globally over the past decade [20]. Carbapenems should be dose adjusted in patients with renal dysfunction. Imipenem use has been associated with central nervous system (CNS) toxicity and should not be used for the therapy of meningitis. Ertapenem has a narrower spectrum of activity (not active against pseudomonas, acinetobacter, enterococcus, and listeria) and can be administered once a day for effective use in the treatment of most ESBL infections.

Monobactams

Aztreonam is a pure gram-negative β-lactam antibiotic with virtually no activity against gram-positive organisms or anaerobes. Aztreonam has been used successfully in patients with penicillin anaphylaxis due to the absence of cross-allergenicity with other β-lactam antibiotics, but ceftazidime allergy is an important exception to this rule because of a shared side chain. Empiric use of aztreonam in penicillin-allergic septic patients is discouraged due to poor efficacy against pseudomonas. For example, pseudomonas sensitivity in our institution to aztreonam is 60%-65%, which is inferior to quinolones. Aztreonam may have a role in the treatment of NDM carbapenemase-producing organisms since it is not degraded by the class B metallo β-lactamases [21].

Broad-Spectrum Fluoroquinolones

Fluoroquinolones are broad-spectrum antibiotics (Table 4) with class-based activity against *pseudomonas* (ciprofloxacin, levofloxacin, and delafloxacin) and *streptococcus pneumoniae* (levofloxacin, moxifloxacin, and delafloxacin). Ciprofloxacin has limited or no activity against gram-positive organisms, and delafloxacin is active against HA-MRSA. Fluoroquinolones are generally not used empirically in the treatment of hospitalized patients with sepsis due to increasing resistance in gram-negative organisms. Delafloxacin has broad antibacterial activity against pathogens causing skin and respiratory infections, but more studies are required for use in the treatment of invasive bloodstream infections [22]. If the isolate is susceptible (e.g., ESBL), fluoroquinolone therapy is an option for treating non-life-threatening infections (e.g., UTI, pneumonia).

**Table 4:** Differences in coverage between various classes of quinolones

Agent	Ciprofloxacin	Levofloxacin	Moxifloxacin	Delafloxacin
Pseudomonas	Moderate	Moderate	Very Poor	Excellent
E. Coli, Klebsiella	Moderate	Moderate	Moderate	Excellent
Strep. pneumoniae	None	Excellent	Excellent	Excellent
Atypical organisms	Some activity	Excellent	Excellent	Excellent
HA- MRSA	None	None	None	Excellent
Anaerobes	None	None	Moderate (not bacteroides)	Some activity in vitro.
Bioavailability	70%	95%	86%	59%
Toxicity to all quinolone classes	QT prolongation, t endinopathy, tendon rupture, peripheral neuropathy (can be permanent). Aortic dissection (avoid use in patients with aortic aneurysms, peripheral vascular disease, Marfan and Ehlers Danlos syndrome). NM blocking activity (avoid in myasthenia gravis – can precipitate crisis). Avoid use in pregnancy and children due to musculoskeletal toxicity. Greater risk of *C. difficile* compared to other antibiotics.			

Broad Spectrum Tetracyclines

Tigecycline is a glycylcycline antibiotic with bacteriostatic activity against many gram-positive pathogens (including MRSA and VRE), anaerobes, and atypical organisms. It also covers some gram negatives (*enterobacter*, *klebsiella*, including ESBL), but has no activity against pseudomonas, *proteus*, *providencia,* and *morganella*. The drug should not be used to treat urinary infections (inadequate drug concentrations) and bacteremias (treatment failures). Several new tetracyclines have been approved for use, including *eravacycline*, *sarecycline,* and *omadacycline,* which may have a role in the treatment of certain multi-drug resistant organisms [23].

Broad-spectrum gram-positive antibiotics

Staphylococcal aureus, enterococcus, and streptococcus pneumoniae comprise the majority of nosocomial gram-positive infections. *S. aureus* releases β-lactamases (penicillinases), but treatment with β-lactam inhibitors is not required, as they can be easily treated with penicillinase-resistant penicillins (e.g., methicillin or nafcillin) or first-generation cephalosporins (e.g., cefazolin). Methicillin is no longer manufactured due to nephrotoxicity, but the terminology of methicillin-susceptible *S. aureus* (MSSA) and methicillin-resistant *S. aureus* (MRSA) remain in use. Nafcillin-resistant *S. aureus* infections are commonly seen in both hospital-acquired (HA-MRSA) and community-acquired (CA-MRSA) strains. Both strains can interchangeably cause community and nosocomial infections including cellulitis, multilobar pneumonia, and endocarditis from cross-transmission. Many CA-MRSA strains are susceptible to tetracyclines, trimethoprim-sulfamethoxazole, and clindamycin (after the D test). CA-MRSA strains may appear susceptible to fluoroquinolones, but resistance develops rapidly during therapy. IV Vancomycin is usually the drug of choice to treat HA-MRSA infections, but other agents (linezolid, telavancin, ceftoraline, and daptomycin) can be used in patients with vancomycin resistance (Table 5).

**Table 5:** Differences in coverage between commonly used gram-positive antibiotics CA-MRSA: Community acquired MRSA; HA-MRSA: Hospital acquired MRSA; VRE: Vancomycin resistant enterococcus; MSSA: Methicillin sensitive staph aureus.

Agent	Clindamycin	Vancomycin	Ceftaroline	Linezolid	Daptomycin
Streptococcus	bacteriostatic	Bactericidal	Bactericidal	Bactericidal	Bactericidal
Enterococcus faecium vs. faecalis	No coverage	Bacteriostatic for E. faecalis	Bacteriostatic for E. faecalis	Bacteriostatic for E. faecium & faecalis	Bactericidal for E. faecium & faecalis
VRE	No coverage	N/A	No coverage	Bacteriostatic	Bactericidal
CA-MRSA	Very good	Excellent	Excellent	Excellent	Excellent
HA-MRSA	No coverage	Excellent	Excellent	Excellent	Excellent
Atypical organisms	Very good	None	None	None	None
Listeria	None	Poor	None	Very good	Poor
MSSA bacteremia	Avoid use	Avoid use (failures reported)	Not enough data	Avoid use (failures reported)	Use in patients with penicillin anaphylaxis
Other gram-positive bacteremias	Avoid use	Very good for Strep and MRSA bacteremia	As good as daptomycin for MRSA bacteremia	Good for Strep and VRE bacteremia, if source is not endocarditis.	Excellent for MRSA and VRE bacteremia and endocarditis.
Pneumonia coverage	Good for CA-MRSA	Good for both CA & HA-MRSA	Good for both CA & HA-MRSA	Good for both CA & HA-MRSA	Avoid use (drug inactivated by surfactant)
Inhibition of bacterial toxin production	Very good	None	None	Very good	None
Anaerobic coverage	Good for most anaerobic infection except bacteroides.	None	Not significant	Good in-vitro coverage observed in studies.	Not significant
Adverse effects	Diarrhea (2-20%), C. Diff (0.1-10%), DRESS syndrome, Sweet syndrome.	Red Man syndrome, Acute kidney injury, Ig E mediated anaphylaxis.	Neutropenia, encephalopathy in patients with renal failure.	Bone marrow suppression, lactic acidosis, ocular toxicity, peripheral neuropathy and serotonin syndrome.	Rhabdomyolysis, Eosinophilic pneumonia, DRESS, peripheral neuropathy, interstitial nephritis and neutropenia.

*Enterococci* (*E. faecium* and *faecalis*) usually rank only second to *staphylococci* in nosocomial gram-positive infections (UTI and bacteremia). Infections due to *E. faecalis* tend to be more virulent (e.g., endocarditis) than infections due to *E. faecium*. There has been a notable rise in *E. faecium* species in recent years, which now accounts for 30%-40% of all enterococcal nosocomial infections. Most clinical isolates of *E. faecium* are resistant to ampicillin and vancomycin. Β-lactam antibiotics (e.g., ampicillin) have only bacteriostatic effect on enterococci requiring aminoglycoside adjunctive therapy for the treatment of endocarditis. Daptomycin has bactericidal activity against enterococci and can be used alone to treat invasive resistant infections. Enterococcal infections can also be due to colonization (e.g., respiratory specimens or urinary catheters), and mere isolation of the organism in cultures does not necessarily require targeted therapy.

Linezolid

Linezolid is uniquely bactericidal against most *streptococcal* species but only bacteriostatic against *staphylococcal* and *enterococcus* species. Linezolid is available in both IV and oral form, which can be used to treat several non-invasive MRSA infections (e.g., cellulitis, diabetic foot infections, pneumonia), including bacteremias (Strep and VRE) resulting from non-endovascular sources (e.g., skin and soft tissue infections). Since the drug binds to bacterial ribosomes and prevents toxin production, it can be used as an alternative to clindamycin for the treatment of serious toxin-producing streptococcal skin infections. Linezolid also has in-vitro activity against certain anaerobes, including *clostridium perfringens*, *clostridium difficile*, *peptostreptococcus,* and *bacteroides fragilis* [24]. It is currently the only agent that carries the FDA indication to treat VRE bacteremia, but most clinicians prefer to use daptomycin due to bactericidal activity. Bone marrow toxicity is an important concern with prolonged use of linezolid, along with other adverse effects (Table 5).

Daptomycin

Daptomycin is a bactericidal drug against most gram-positive organisms, including enterococcus, but it has decreased activity against listeria monocytogenes and actinomyces species. Daptomycin is inactivated by alveolar surfactants and, therefore, should not be used to treat pneumonia. Daptomycin can be used in the treatment of skin, soft tissue, bone/joint, and urinary tract infections but penetrates poorly into the CSF. In patients with refractory MRSA bacteremia, despite the use of daptomycin, adjunctive ceftaroline therapy can be used for synergistic bactericidal activity [25]. Daptomycin dosing for bloodstream infections is usually 6 mg/kg IV once daily, but higher doses (8-12 mg/kg) may be warranted in critically ill patients [26]. Daptomycin-induced myopathy may develop with or without symptoms, and increased risk is seen in patients with obesity and concomitant use of statins. Creatinine kinase levels should be checked frequently during therapy, along with patient screening for the development of muscle pain or weakness.

Conditions that may not respond to broad-spectrum antibiotic therapy

MSSA Bacteremia

Vancomycin is slowly bactericidal (around 32 hours) to methicillin-sensitive staphylococci and is considered inferior to anti-staphylococcal penicillins (e.g., nafcillin) and first-generation cephalosporins (e.g., cefazolin), where a cidal effect seen within four hours [27]. Both cefepime and carbapenems have excellent in-vitro coverage towards MSSA, but treatment failures have been reported with piperacillin/tazobactam. For the treatment of MSSA bacteremia and infective endocarditis, nafcillin is the preferred agent to treat MSSA bacteremia over cefazolin in patients with high inoculum effect (significant worsening of MIC at higher bacterial inoculum samples), which can lead to treatment failures [28]. Due to the difficulties in administration of nafcillin (every four hours or continuous dosing), the volume of fluid administered, and a higher incidence of renal failure, treatment can be switched in stable patients to cefazolin for the remainder of therapy. For the treatment of patients with a history of documented penicillin anaphylaxis, daptomycin can be used to treat MSSA bacteremia.

Skin and Soft Tissue Infections (SSTI)

The emergence of community-associated MRSA strains (typically presents with purulence) has greatly influenced the selection of empirical antibiotic therapy for SSTI. Clindamycin resistance is growing among *S. Aureus* (around 65% sensitive in our institution) and physicians are encouraged to review the local resistance patterns before choosing a monotherapy agent for cellulitis. For the treatment of most patients with simple cellulitis (non-purulent), first-generation cephalosporins (e.g., cephalexin) are recommended. In patients with purulent SSTI, they can be combined with other agents (e.g., trimethoprim-sulfamethoxazole, tetracyclines) with activity against community-acquired MRSA infection. Empiric coverage for gram-negative organisms (including pseudomonas) and anaerobes is sometimes needed in patients with poorly controlled diabetes presenting with severe SSTI. In hospitalized patients with severe necrotizing soft tissue infections and toxic shock syndrome, bacterial protein synthesis inhibitors (e.g., clindamycin or linezolid) can be added for antitoxin effects [29]. Necrotizing SSTIs often require aggressive surgical debridement, broad-spectrum antimicrobials, and intensive care. 

Combination antimicrobial therapy for resistant bacterial infections

*Pseudomonas aeruginosa* is one of the most common gram-negative nosocomial infections that is associated with high mortality. In most cases, the use of a single β-lactam agent over a combination therapy is preferred when treating serious *P. aeruginosa* infections, including bacteremia [30-31]. Combination therapy with two agents from different classes with in vitro activity against *P. aeruginosa* (i.e., β-lactam-aminoglycoside or β-lactam-quinolone combinations) should be reserved in patients with especially high mortality (e.g., septic shock, endocarditis, neutropenia and burn injuries). For example, pseudomonas sensitivity in our institution for traditional β-lactam agents (e.g., cefepime, piperacillin-tazobactam, and meropenem) is between 83%-85% and it increases to >99% after the addition of aminoglycosides.

Synergistic double or triple antibiotic combinations that include an aminoglycoside, ampicillin/sulbactam, carbapenem, aztreonam, tigecycline, colistin, fosfomycin or rifampin have been used to treat multidrug-resistant *acinetobacter* species, staphylococcal prosthetic valve endocarditis and carbapenemase-producing Enterobacteriaceae.

Double β- lactam therapy is indicated empirically to treat listeria/pneumococcal meningitis (ampicillin + ceftriaxone), synergistically for enterococcus endocarditis (ampicillin + ceftriaxone) and as a salvage therapy (anti-staphylococcal penicillin + ertapenem) for refractory MSSA bacteremia [32]. Double β-lactam therapy has a better safety profile when compared to β-lactam plus aminoglycoside combinations [33].

Approach to septic patients who fail the initial therapy

Patients having persistent hypotension despite adequate fluid resuscitation and antimicrobial treatment should be evaluated for transfer to the ICU for initiating vasopressor (norepinephrine) therapy in early septic shock and inotropic (dobutamine) therapy as needed in late septic shock with diminished cardiac output. Adjunctive therapy with IV hydrocortisone (around five days) can be used in selected patients with hypocortisolemia (serum cortisol <15mcg/dL) to treat relative adrenal insufficiency. Corticosteroid therapy may result in faster resolution of shock with no or minimal mortality benefit. Routine administration of empirical antifungal therapy is not generally warranted in critically ill patients, but may be considered in neutropenic patients [34]. Early consultation with infectious disease specialist in septic patients has been associated with a 40% reduction in mortality [35]. 

Approach to septic patients who respond to the initial therapy

Once the patient appears hemodynamically stable and defervesced with improving leukocytosis, attention should be directed towards the de-escalation of antibiotics based on the radiologic studies and culture results. It is easy to narrow the antimicrobial therapy in septic patients with positive cultures based on susceptibilities. In patients with culture-negative sepsis (around 50% cases), de-escalation of empiric therapy requires clinical judgment and close monitoring of the patient while attempting the removal of one agent at a time (vancomycin is typically discontinued first). The duration of antibiotics should be individualized and for most infections (e.g., pneumonia, UTI, meningitis), the duration of therapy is typically seven to 10 days. Unexplained bacteremias with no endovascular focus (e.g., MSSA, MRSA) can be treated with IV antibiotics for 14 days. Longer courses (6-8 weeks) of IV antibiotics are required to treat endocarditis and osteomyelitis. Patients on long-term hemodialysis may receive certain antibiotics (vancomycin, cefazolin, cefepime, ertapenem, and daptomycin) thrice weekly post-hemodialysis, which precludes the need for the placement of indwelling catheters (e.g., PICC line). Patients with gram-negative bacteremias from UTI and gram-positive bacteremias from pneumonia or cellulitis can be safely treated with oral antibiotics.

## Conclusions

Sepsis should be considered a medical emergency, and appropriate and timely empiric antibiotic treatment is one of the cornerstones of therapy to prevent high mortality. In septic patients, IV fluid administration must be guided using clinical targets, including mean arterial pressure of 60 to 70 mmHg and urine output ≥0.5 mL/kg/hour and serum lactate should be followed (e.g., every six hours) until there is a definitive clinical response. Empirical beta-lactam-based broad-spectrum antibiotic regimens must be selected based on the specific sites of infection and the local antimicrobial resistance patterns. In patients with penicillin allergy/anaphylaxis, either cefepime or meropenem should be the empiric drug of choice instead of aztreonam due to widespread bacterial resistance. Following initial investigations and empiric antimicrobial therapy, further efforts aimed at identifying and controlling the source(s) of infection should be performed. Culture-negative sepsis is prevalent in tertiary care centers, and the possibility should be considered in septic patients with negative cultures. Attempts to de-escalate the initial broad-spectrum antibiotics after 48 hours will help to reduce the spread of antibiotic resistance. Shortened courses of antimicrobials must be considered for most patients, with the important exception of invasive infections of the bloodstream and bone. Patients should be closely monitored for antibiotic-associated adverse events like renal failure and encephalitis, and alternative agents must be instituted. Both VRE and ESBL commonly colonize the indwelling urinary catheters, and most cases, do not need antibiotic therapy.
